# Surgical and functional outcomes of robot-assisted versus laparoscopic partial nephrectomy with cortical renorrhaphy omission

**DOI:** 10.1038/s41598-022-17496-2

**Published:** 2022-07-29

**Authors:** Masashi Kubota, Toshinari Yamasaki, Shiori Murata, Yohei Abe, Yoichiro Tohi, Yuta Mine, Hiroki Hagimoto, Hidetoshi Kokubun, Issei Suzuki, Naofumi Tsutsumi, Koji Inoue, Mutsushi Kawakita

**Affiliations:** Department of Urology, Kobe City Medical Centre General Hospital, 2-1-1 Minatojima-Minamimachi, Chuo-ku, Kobe, 650-0047 Japan

**Keywords:** Urology, Surgical oncology, Nephrons

## Abstract

To evaluate the surgical and functional outcomes between robot-assisted (CRO-RAPN) vs. laparoscopic (CRO-LPN) methods of cortical-renorrhaphy-omitting partial nephrectomy. Between July 2012 and June 2020, patients with localized clinical T1-2 renal masses who underwent CRO-RAPN or CRO-LPN were reviewed. The outcomes of the two groups were compared using propensity-score matching. Trifecta was defined as negative surgical margin, warm ischemic time < 25 min, and absence of complications of Clavien-Dindo grade III or more until three months postoperatively. The preservation rate of the estimated glomerular filtration rate (eGFR) was evaluated at six months postoperatively. Among 291 patients (CRO-RAPN, n = 210; CRO-LPN, n = 81) included in the study, 150 matched pairs of patients were analyzed. Compared to the CRO-LPN group, the CRO-RAPN group was associated with shorter warm ischemic time (13 min vs. 20 min, *P* < 0.001), shorter total operation time (162 min vs. 212 min, *P* < 0.001), less estimated blood loss (40 mL vs. 119 mL, *P* = 0.002), lower incidence of overall complications (3% vs. 16%, *P* = 0.001), higher preservation rate of eGFR at six months postoperatively (93% vs. 89%, *P* = 0.003), and higher trifecta achievement rate (84% vs. 64%, *P* = 0.004). CRO-RAPN contributed to shorter warm ischemic time, less blood loss, fewer complications, and higher preservation of renal function, all of which allowed this technique to achieve a higher rate of trifecta compared to CRO-LPN.

## Introduction

Partial nephrectomy (PN) is the gold standard definitive therapy for T1 renal masses with surgical indication^1,2^. Robot-assisted partial nephrectomy (RAPN) and laparoscopic partial nephrectomy (LPN) are minimally invasive PN with recently expanded indications for complex and challenging renal tumors^3,4^. In early LPN series, suturing renorrhaphy of the renal cortex layer was considered an indispensable procedure to ensure hemostasis and closure of the urinary collecting system so that postoperative complications could be avoided. In recent years, concerns with excessive renorrhaphy have emerged since an injured vascularized parenchyma prevents the preservation of postoperative renal function^5,6^. While a consensus regarding the optimal renorrhaphy technique for postoperative renal function preservation has not yet been established, the single-layer renorrhaphy technique has been favorably considered for postoperative renal function compared to the double-layer technique^7,8^. Furthermore, the omission of a cortical suture layer represented an ideal approach to reduce complication risk and preserve healthy renal parenchyma^5,9–12^. However, the optimal platform to safely perform this challenging procedure has not been established. Previous studies have indicated that RAPN is more favorable than LPN in terms of renal functional preservation outcomes, shorter duration of hospital stay, and shorter warm ischemia time^13–15^. However, these advantages of RAPN compared to LPN are limited only among the conventional double-layer renorrhaphy technique. Therefore, this study aimed to evaluate the surgical and functional outcomes of cortical-renorrhaphy-omitting RAPN (CRO-RAPN) in comparison to those of cortical-renorrhaphy-omitting LPN (CRO-LPN).

## Patients and methods

### Study cohort and design

This retrospective study included prospectively maintained data from an institutional database and was approved by our institutional review board. All registered patients in the database, who were diagnosed with clinical T1-2 renal tumors and underwent PN between July 2012 and June 2020, were screened for possible retrospective analysis. The exclusion criteria were as follows: patients who underwent open PN, solitary kidney, or recurrent and distant metastasis; presence of bilateral or multifocal tumors; receiving intraoperative cortical-layer renorrhaphy due to (1) previous decision to undergo preoperative cortical renorrhaphy as a result of tumor complexity or (2) involvement in other clinical trials with intraoperative cortical-layer renorrhaphy requirement. One group of patients underwent RAPN (CRO-RAPN group), and the other group received LPN (CRO-LPN group). Patient characteristics and clinical outcomes were compared between these two groups. RAPN vs. LPN indications were distinguished by the operation date. Specifically, all patients who received surgical procedures after April 2016 were switched from LPN to RAPN since RAPN was approved by the Ministry of Health, Labor and Welfare and covered by the national health insurance.

To adjust for differences in baseline characteristics between CRO-RAPN and CRO-LPN patients, propensity-score matching was conducted. The propensity scores were calculated by logistic regression analysis with the odds of undergoing LPN as the dependent variable and following independent variables: age (years), sex (male vs. female), body mass index (kg/m^2^), American Society of Anesthesiologists Physical Status (ASA PS) (grades 1–2 vs. 3 or higher), chronic kidney disease, Kidney Disease Improving Global Outcomes (KDIGO) classification^14^ (grades 1–2 vs. 3a–3b vs. 4–5), tumor side (right vs. left), tumor size (mm), R.E.N.A.L. nephrometry score (4–6 vs. 7–9 vs. 10–12), and off-clamp procedure (on-clamp vs. off-clamp). Patient characteristics of two groups were matched in a one-to-one ratio according to the propensity score. A caliper width of 0.2, for the standard deviation, was applied.

### Surgical procedure

**Figure 1:**
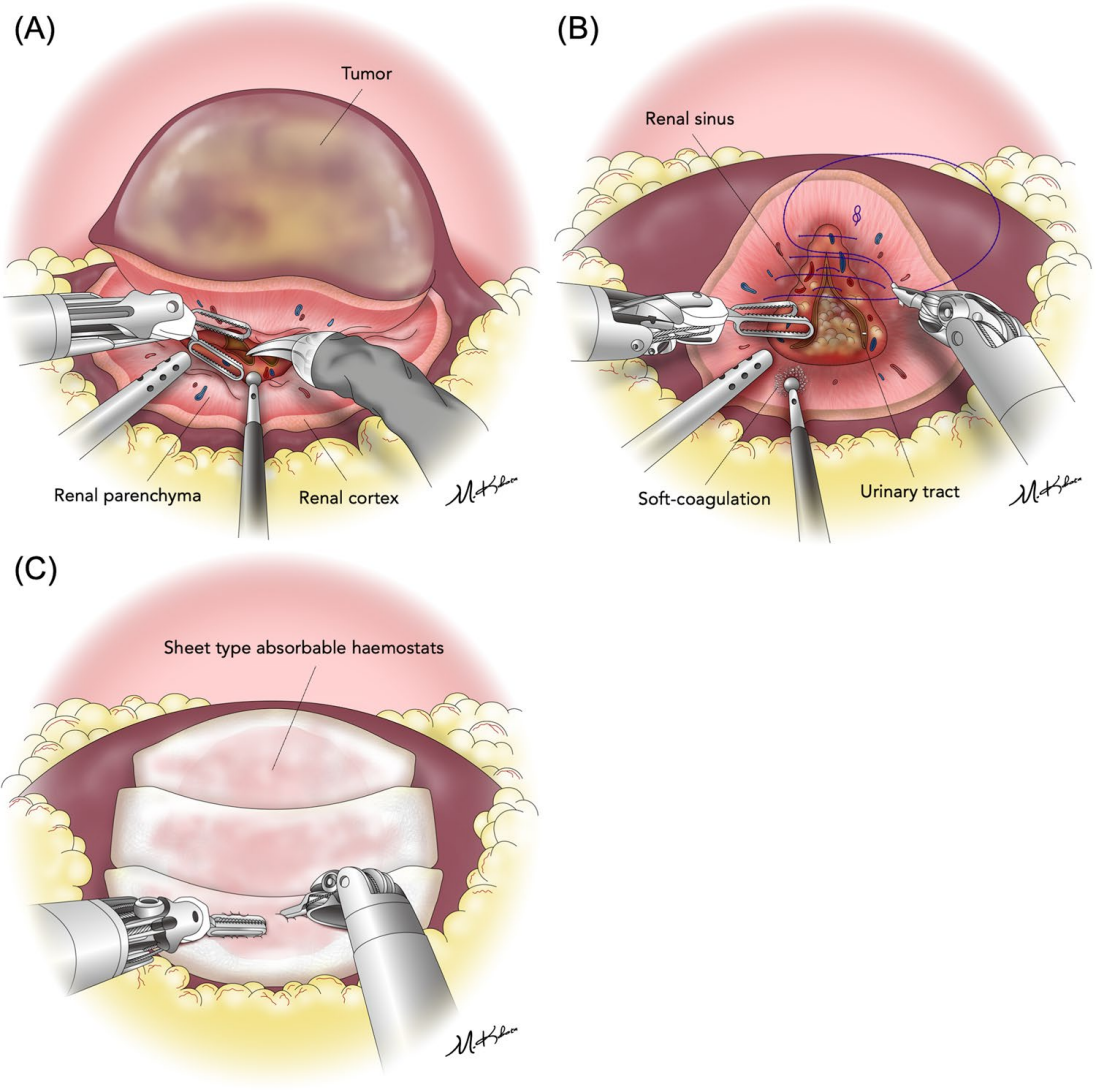
The cortical-renorrhaphy-omitting surgical procedure during partial nephrectomy in this study. In cases without intervention of the renal sinus or urinary tract, the inner suture was omitted and the operation was completed without suturing (**a, c**). If the renal sinus or urinary tract was opened, an inner suture was performed (**b**)**.** (**a**) Tumor resection technique: the renal parenchyma was bluntly dissected along with fiber lines. Bleeding from the cortical or parenchymal vasculature was controlled by soft coagulation. (**b**) Inner suture technique: opened renal sinuses or urinary collecting systems were closed by running a suture in the parenchymal layer with shallow stitch by 3–0 V-LOC 180 V20 (Covidien; New Haven, CT, USA). (**c**) Renal cortical hemostasis: cortical renorrhaphy was omitted, and the sheet-type absorbable hemostats (TachoSil; CSL Behring) were onlayed on the tumor bed.

The omission of cortical renorrhaphy during LPN and RAPN was performed as the standard procedure at our institution. However, some complex cases with entirely endophytic tumors were excluded the study and underwent cortical-layer renorrhaphy because the deep and narrow resection surface of such tumors was difficult to be effectively stitched within the parenchymal layer limit. Intraoperative procedures were standardized and not modified for each group. LPN was performed using four or five ports and RAPN was performed using a four-arm da Vinci Si, X, Xi®–system (Intuitive Surgical, Sunnyvale, CA, USA) as previously reported^12^. An intraoperative ultrasonography probe (L43K or L51K in RAPN, Noblus in LPN; Hitachi, Tokyo, Japan) was routinely used to identify the tumor location, size, depth, and flow. With ultrasound guidance, the resection margin was delineated. The main renal artery was completely clamped using laparoscopic bulldog clamps, and the venous system was unclamped. The off-clamp technique was indicated for patients with preoperative computed tomography (CT) findings of externally budding renal tumors, which were distant from the renal sinus. Tumor resection and hemostasis are shown in Fig. 1. The renal capsule and cortical layer were cut with a margin of approximately 5–10 mm for LPN and 0–5 mm for RAPN. Upon reaching the peritumoral parenchymal layer, the tumor was bluntly dissected along with fiber lines of the renal parenchyma and resected with a thin margin to preserve the normal parenchyma as much as possible. During tumor resection, suction and soft coagulation (VIO 300D; ERBE Elektromedizin GmbH) of a ball-type electrode were performed by the assistant to control bleeding. In cases without intervention of the renal sinus or urinary tract, the inner suture was omitted, and the operation was completed without suturing. Single-layer inner running sutures (15 cm 3–0 V-Loc 180 V20; Covidien, New Haven, CT, USA) were performed only when entry into the collecting system or renal sinus was detected. For the inner sutures, the running needle was pierced at the inner edge of the renal parenchyma, and only a minimal amount of thin renal sinus tissue was sutured for closure. After inner suturing, the clamps (if used) were removed. After soft coagulation, absorbable hemostats (TachoSil; CSL Behring) were placed on the resection bed and manually pressed for a few minutes. A single expert surgeon who had performed over 300 pure LPN supervised a team of surgeons with previous training of the surgeries involved in this study. Routine CT scans were scheduled between postoperative days 3 and 7.

### Data collection for clinical and surgical outcomes

Patient data extracted from the prospective database included age, sex, body mass index, estimated glomerular filtration rate (eGFR), oncological background (clinical TNM stage, tumor side, tumor size, and R.E.N.A.L nephrometry score), perioperative information (ASA PS), total operation time, warm ischemic time, off-clamp technique, estimated blood loss, transfusions, surgeon, length of hospital stay, readmission, in-hospital complications, and Clavien-Dindo grade^16^. Regarding post-discharge complications, retrospective data were collected by reviewing the outpatient medical records.

The primary outcome was the achievement rate of trifecta, defined as a negative surgical margin, warm ischemic time < 25 min, and no complications of Clavien-Dindo grade III or higher until three months postoperatively^17^. The secondary outcomes were as follows: total operation time, median warm ischemic time, intraoperative estimated blood loss, preservation rate of eGFR at six months postoperatively, stage upgrade of chronic kidney disease KDIGO grade^18^, and the incidence of overall complications (Clavien-Dindo grade II or higher) at three months postoperatively. Bleeding-related complications included secondary procedures due to postoperative bleeding, transfusion, or intervention for postoperative hematoma infection.

### Statistical analysis

A standard statistical software package (JMP®, ver. 13; SAS Institute, Chicago, IL, USA) was used for statistical analyses. The Mann–Whitney U and Chi-squared tests were used to determine the statistically significant differences between two groups in the univariate analysis. Statistical significance was set at *P* < 0.05.

### Ethical approval

All procedures involving human participants in the present study were conducted in accordance with the ethical standards of the institutional research committee and with the 1964 Helsinki Declaration and its later amendments or comparable ethical standards. The study protocol was approved by Kobe City Medical Centre General Hospital institutional review board (IRB No. zn210405). The need for informed consent was waived by the institutional review committee due to the retrospective nature of the study.

## Results

**Figure 2:**
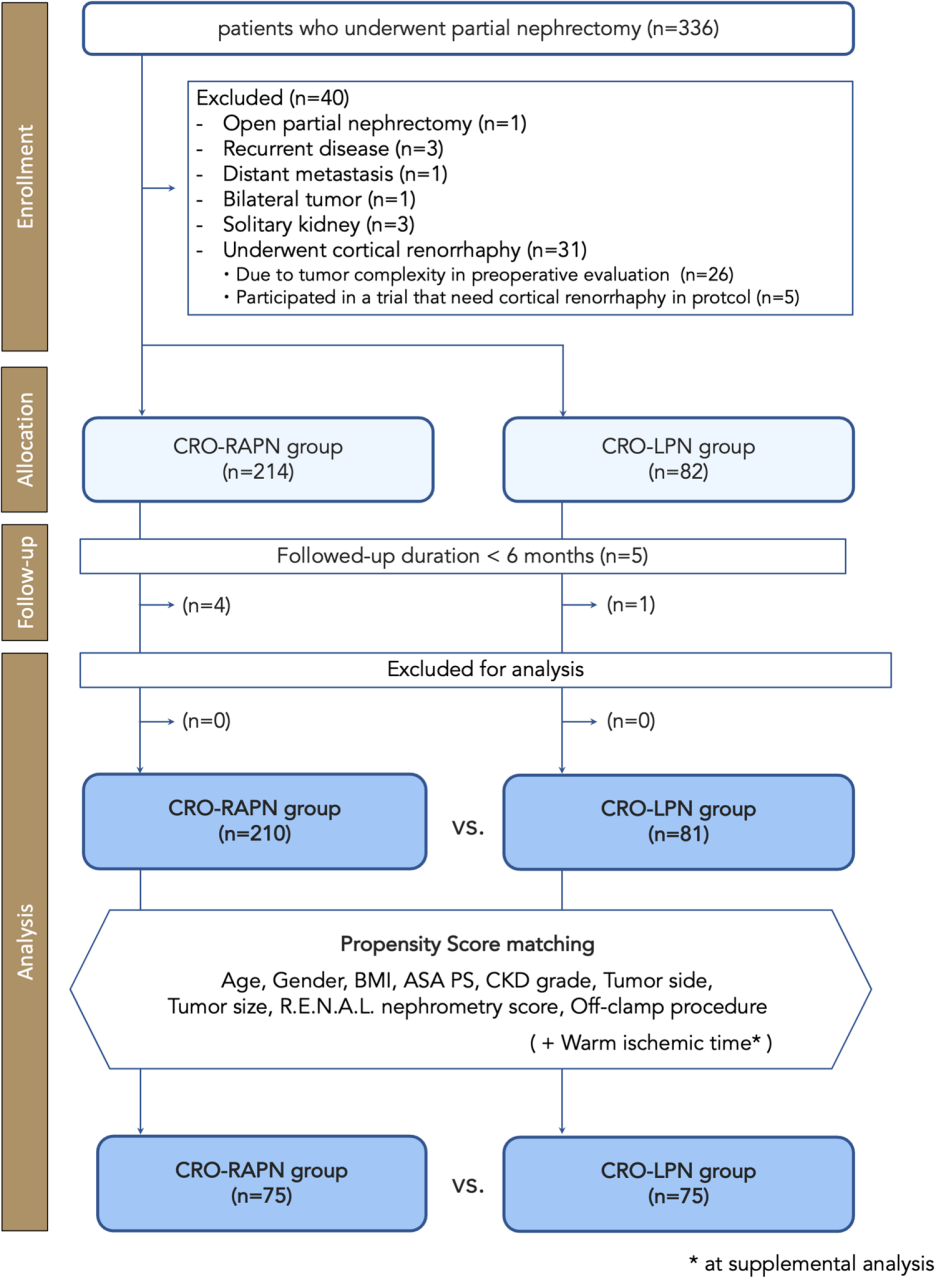
Flow diagram of patient enrolment.

A flow diagram of the study is shown in Fig. 2. Among the 336 consecutive patients included in the screening process, 40 patients were removed based on the exclusion criteria (20 with LPN and 6 with RAPN in the initial phase decided to undergo preoperative cortical renorrhaphy due to tumor complexity, 5 participated in a trial with cortical renorrhaphy requirement). Five patients were lost during follow-up at six months postoperatively. Consequently, 291 patients, including 210 patients in the CRO-RAPN group and 81 patients in the CRO-LPN group, were included in this study. All patients were followed up for at least six months postoperatively and available for data collection with no missing data for the required parameters.

As shown in Table 1, CRO-RAPN group had significantly poorer eGFR values (67 mL/min/1.73 m^2^ vs. 73 mL/min/1.73 m^2^, *P* = 0.040) and higher R.E.N.A.L. nephrometry scores (≥ 7: 55% vs. 41%, *P* = 0.047) compared to the CRO-LPN group. The off-clamp procedure was indicated more frequently for CRO-LPN patients than those in the CRO-RAPN group (23% vs. 6%, *P* < 0.001). There were no significant differences in other baseline characteristics or surgical procedures between two groups. After propensity-score matching, 75 matched pairs were available for the final analysis, which showed no significant differences in any of the baseline characteristics between the CRO-RAPN and CRO-LPN groups.

**Table 1:** Patient characteristics of the CRO-RAPN and CRO-LPN groups before and after propensity-score matching.

Parameters	Before propensity-score matching			After propensity-score matching		
	CRO-RAPN^f^ group	CRO-LPN^g^ group	*p*-value	CRO-RAPN group	CRO-LPN group	*p*-value
Number of patients	210	81		75	75	
Median age, years (IQR^a^)	67 (57–75)	68 (54–76)	0.79	67 (57–74)	68 (52–75)	0.77
Male sex, n (%)	128 (61)	50 (62)	0.51	49 (65)	46 (61)	0.37
Median BMI^b^, kg/m^2^ (IQR)	23.9 (21.5–26.4)	24.3 (21.8–25.9)	0.96	23.6 (20.5–26.2)	24.3 (22.0–26.0)	0.39
ASA PS^c^ 3 or more, n (%)	25 (12)	8 (10)	0.40	11 (15)	7 (9)	0.23
eGFR^d^, mL/min/1.73m^2^ (IQR)	67 (55–77)	73 (56–85)	0.040	68 (59–81)	73 (56–85)	0.31
**KDIGO CKD**^**e**^**grade, n (%)**						
Grade 1 or 2 (60 mL/min/1.73 m^2^, or more)	143 (68)	60 (74)	0.55	56 (74)	56 (74)	1.0
Grade 3a or 3b (30–59 mL/min/1.73 m^2^)	58 (28)	19 (23)		17 (23)	17 (23)	
Grade 4 or 5 (29 mL/min/1.73 m^2^, or less)	9 (4)	2 (2)		2 (3)	2 (3)	
Right side tumor, n (%)	97 (46)	41 (51)	0.29	42 (56)	36 (48)	0.21
Tumor size, mm (IQR)	30 (22–39)	29 (23–37)	0.84	25 (18–39)	29 (23–37)	0.21
**Clinical T stage**						
T1a	159 (75)	64 (79)	0.59	58 (77)	60 (80)	0.81
T1b	44 (22)	16 (20)		15 (20)	14 (19)	
T2	7 (3)	1 (1)		2 (3)	1 (1)	
**R.E.N.A.L. nephrometry score, n (%)**						
4–6 (Low)	95 (45)	48 (59)	0.047	47 (63)	44 (59)	0.68
7–9 (Intermediate)	89 (42)	29 (36)		26 (35)	27 (36)	
10–12 (High)	26 (13)	4 (5)		2 (3)	4 (5)	
Off-clamp procedure, n (%)	13 (6)	19 (23)	< 0.001	12 (16)	13 (17)	0.50

^a^interquartile range; ^b^body mass index; ^c^American Society of Anesthesiologists Physical Status classification; ^d^estimated glomerular filtration rate; ^e^chronic kidney disease; ^f^cortical-renorrhaphy-omitting robot-assisted partial nephrectomy; ^g^cortical-renorrhaphy-omitting laparoscopic partial nephrectomy.

The perioperative and histopathological outcomes before and after propensity-score matching between the CRO-RAPN and CRO-LPN groups are shown in Table 2 and Supplemental Table 1. As the primary outcome, the trifecta achievement rates of the CRO-RAPN and CRO-LPN groups before and after propensity-score matching was 79% vs. 67% (*P* = 0.027), and 84% vs. 64% (*P* = 0.004), respectively. Moreover, the median total operation time (162 min vs. 212 min, *P* < 0.001), median warm ischemic time (13 min vs. 20 min, *P* < 0.001), median estimated blood loss (40 mL vs. 119 mL, *P* = 0.002), incidence of negative three-month overall complications (97% vs. 84%, *P* = 0.001), median postoperative six-month decrease in eGFR (5 mL/min/1.73 m^2^ vs. 7 mL/min/1.73 m^2^, *P* = 0.002), achievement rate of at least 90% eGFR preservation (67% vs. 48%, *P* = 0.016), and the median six-month preservation rate of eGFR (93% vs/ 89%, *P* = 0.003) in the CRO-RAPN group were also significantly better than those in the CRO-LPN group after propensity-score matching.

**Table 2:** Surgical outcomes and complications of the CRO-RAPN and CRO-LPN groups before and after propensity-score matching.

Parameters	Before propensity-score matching			After propensity-score matching		
	CRO-RAPN^c^ group	CRO-LPN^d^ group	*p*-value	CRO-RAPN group	CRO-LPN group	*p*-value
Number of patients	210	81		75	75	
Median total operation time, minutes (IQR^a^)	169 (144–196)	212 (183–238)	< 0.001	162 (139–181)	212 (184–237)	< 0.001
Median warm ischemic time, minutes (IQR)	16 (11–23)	20 (16–28)	< 0.001	13 (10–21)	20 (16–28)	< 0.001
Median blood loss, mL (IQR)	40 (15–90)	119 (40–300)	< 0.001	40 (10–90)	119 (40–300)	0.002
Transfusion, n (%)	2 (1)	3 (4)	0.014	1 (1)	3 (4)	0.31
Underwent inner suture, n (%)	134 (64)	51 (63)	0.50	41 (55)	50 (67)	0.091
Urinary tract entry, n (%)	111 (53)	39 (48)	0.28	31 (41)	38 (51)	0.16
Positive surgical margin, n (%)	7 (3)	6 (7)	0.12	2 (3)	6 (8)	0.14
Hospital stay, days (%)	5 (4–6)	6 (5–7)	0.027	5 (5–6)	6 (5–7)	0.13
**3-month postoperative complication, n (%)**						
Overall (Clavien-Dindo grade ≥ II)	7 (3)	12 (15)	< 0.001	1 (3)	12 (16)	0.001
High-grade (Clavien-Dindo grade ≥ III)	4 (2)	3 (4)	0.3	1 (1)	3 (4)	0.31
Bleeding related	4 (2)	7 (9)	0.013	1 (1)	7 (9)	0.032
Urine leakage	2 (1)	0 (0)	0.52	0 (0)	0 (0)	1
Mortality	0 (0)	0 (0)	1.0	0 (0)	0 (0)	1.0
**6-month preservation rate of eGFR**^**b**^						
Median, % (IQR)	92 (85–100)	88 (81–95)	0.005	93 (87–100)	89 (81–95)	0.003
≥ 90%, n (%)	129 (61)	37 (46)	0.011	50 (67)	36 (48)	0.016
≥ 80%, n (%)	185 (88)	68 (84)	0.23	66 (88)	63 (84)	0.16
≥ 70%, n (%)	206 (98)	77 (95)	0.15	74 (99)	71 (95)	0.18
Median 6-month decrease of eGFR, mL/min/1.73 m^2^ (IQR)	5 (0–10)	7 (4–12)	0.001	5 (0–8)	7 (4–12)	0.002
Upstaging of chronic kidney disease, n (%)	50 (24)	22 (27)	0.33	17 (23)	20 (27)	0.35
Trifecta achievement rate, n (%)	165 (79)	54 (67)	0.027	63 (84)	48 (64)	0.004

^a^interquartile range; ^b^estimated glomerular filtration rate; ^c^cortical-renorrhaphy-omitting robot-assisted partial nephrectomy; ^d^cortical-renorrhaphy-omitting laparoscopic partial nephrectomy.

Before propensity-score matching, two (1%) patients underwent transcatheter arterial embolization due to postoperative bleeding in the CRO-RAPN group. Of these, one patient was admitted for postoperative urosepsis and a pseudoaneurysm was detected on the tumor bed on emergency CT. Bleeding in the mesentery of the sigmoid colon was detected in the other patient due to intraoperative injury of the branch of the inferior mesenteric artery. In the CRO-LPN group, one patient required transcatheter arterial embolization due to hemorrhagic shock from postoperative tumor bed bleeding. No other patients showed active bleeding or pseudoaneurysm on routine postoperative CT. In the post-matching cohort, seven patients (9%) experienced bleeding-related complications in the CRO-LPN group. Among these, one (1%) had postoperative bleeding, 3 (4%) needed transfusion due to intraoperative bleeding, and 3 (4%) required intravenous antibiotics due to infection of the perirenal hematoma. However, only one patient (1%) needed intervention due to bleeding-related complications in the CRO-RAPN group, and this incidence was significantly lower than that of LPN group (*P* = 0.032).

To adjust for the impact of renal ischemia on the functional outcomes^19–21^, additional propensity-score matching analysis of warm ischemic time was added as another background factor to the eleven factors of the prior analysis. The functional outcomes between the CRO-RAPN and CRO-LPN groups were re-compared (Supplemental Tables 2 and 3). Significant differences between the two groups in the median 6-month preservation rate and the decrease of eGFR were confirmed in this warm-ischemic-time-adjusted analysis (*P* = 0.031 and 0.028, respectively). Furthermore, the delta variations of eGFR at 3 and 6 months postoperatively in CRO-RAPN group were superior to those of the CRO-LPN group after propensity-score matching of these twelve parameters (*P* = 0.018) (Supplemental Fig. 1).

## Discussion

In this study, CRO-RAPN patients had a shorter intraoperative warm ischemic time and lower incidence of complications than CRO-LPN patients. These results contributed to the improvement in trifecta achievement rate for the CRO-RAPN group. Moreover, CRO-RAPN patients also had higher preservation rate of postoperative renal function than CRO-LPN patients. These findings were confirmed after propensity-score matching with adjustment for patient characteristics between the two groups. Importantly, while prior literature reviews have shown that RAPN is superior to LPN in terms of estimated blood loss, warm ischemic time, and renal function preservation, at six months postoperatively^13–15^, our study provided an update for these results with the recent broad introduction of the cortical-renorrhaphy-omitting (CRO) procedures in PN. Recently, the superiority of RAPN to pure LPN was also demonstrated by Bertolo et al. in a cohort of patients who underwent the off-clamp technique^22^. Since the cohort in this study also included many patients who underwent single-layer renorrhaphy, their results were consistent with our findings.

Robotic instruments enhanced the three-dimensional visualization of the operative field and increased the degree of freedom of powered wrists. This innovation allows precise closure of the resected renal sinus or urinary collecting system by inner suture that involves minimal volume of renal parenchyma. Even in cases of small renal tumors that do not require an inner suture, bleeding control during resection with shorter warm ischemic times of robots may provide PN with accurate surgical margins and minimal volume loss of renal parenchyma. While this result was also feasible with LPN, our findings suggested that achieving such ideal PNs is easier with RAPN. Among the trifecta outcomes, postoperative complications was significantly improved in the CRO-RAPN group compared to the CRO-LPN group. Notably, the lower incidence of bleeding-related complications in the CRO-RAPN patients suggested an excellent control of bleeding in this group, which could have ultimately contributed to the improvement of the overall complication rate.

In this study, trifecta achievement rate was utilized as the primary outcome for the comprehensive measurement of the surgical quality and safety. Although several previous studies have proposed different definitions of trifecta of PN^17,23–25^, the definition with the inclusion of the warm ischemic time as a trifecta factor was considered appropriate for the evaluation of the challenging CRO technique during PN. Based on previous studies, the estimated goal for achievement rate of trifecta of RAPN is 68.0–82.6%^17,23–25^. Since the trifecta achievement rates of the RAPN and LPN groups after propensity-score matching in this study were 84% and 64%, respectively, our results show that a sufficiently high achievement rate of trifecta with RAPN and LPN is achievable, even with the CRO procedure. Among previous studies, Hung et al.^24^ first proposed over 90% preservation of eGFR at six months postoperatively as an element of trifecta. However, due to the expanded indication of PN for larger renal tumors, it has become increasingly difficult to achieve such a high eGFR preservation rate due to the large renal volume deficit. In the additional propensity-score matching analysis with adjustment for warm ischemic time in this study, the difference in the preservation rate of eGFR between robot-assisted and laparoscopic partial nephrectomies was confirmed. This result suggests that the transition from laparoscopic to robot-assisted partial nephrectomy showed a positive impact on postoperative renal function, even if the factor of warm ischemic time was excluded. Moreover, the results of multivariate analysis of variance revealed that CRO-RAPN was superior to CRO-LPN in the time-dependent delta variation of eGFR. These results supported the fact that RAPN is a more ideal platform to preserve postoperative eGFR compared to LPN in the case of PN omitting cortical renorrhaphy.

Our study has some limitations. First, this retrospective study involved a patient cohort from a single institution, with a limited number of patients in the final analysis. Because we selected a composite outcome, such as trifecta, as the primary outcome of this study, factors directly involved in each outcome could not be evaluated. In other words, whether decreased blood loss or shortened warm ischemic time directly contributed to the preservation of renal function remains unknown. In addition, significant concerns about the learning curve^26^ could not be disregarded because CRO-RAPN or CRO-LPN indications were divided by the date of the institutional introduction of robotic systems in this study. A randomized prospective setting would be an important study design to address potential differences in surgical skills, and related issues. Furthermore, data on the volume of the resected renal parenchyma or contact surface area could not be included in the analysis due to statistical complexity. Although very few cases, LPN for T2 renal tumors included rare scenarios. The resection strategy was mainly used for early LPN cases, while enucleoresection^27,28^ was mainly used in latter RAPN cases. In this regard, the parameters of the surface-intermediate-base margin score^27,28^ provide an estimated value for parenchyma volume loss, which could address this problem in future studies. Despite of these limitations, the present study showed that CRO-RAPN can be performed safely with a higher trifecta achievement rate compared to CRO-LPN. During CRO procedure, RAPN allowed the achievement of shorter warm ischemic time, less blood loss, fewer complications, and decreased loss of renal function compared to LPN.

## Supplementary Information

Supplementary Information.

## Abbreviations

ASA PS: American society of anesthesiologists physical status
CRO: Cortical-renorrhaphy-omitting
CT: Computed tomography
eGFR: Estimated glomerular filtration rate
KDIGO: Kidney disease improving global outcomes
LPN: Laparoscopic partial nephrectomy
PN: Partial nephrectomy
RAPN: Robot-assisted partial nephrectomy
