# Supplementary material for: Surgical and functional outcomes of robot-assisted versus laparoscopic partial nephrectomy with cortical renorrhaphy omission
Source: Sci Rep. 2022 Jul 29;12:13000. doi: 10.1038/s41598-022-17496-2 (PMC9338244; doi:10.1038/s41598-022-17496-2)
Supplement: Supplementary file 1 — Supplementary Information. [file 41598_2022_17496_MOESM1_ESM.docx]

**Surgical and Functional Outcomes of Robot-Assisted versus Laparoscopic Techniques for Partial Nephrectomy with Cortical Renorrhaphy Omission**

Masashi Kubota, Toshinari Yamasaki, Shiori Murata, Yohei Abe, Yoichiro Tohi, Yuta Mine, Hiroki Hagimoto, Hidetoshi Kokubun, Issei Suzuki, Naofumi Tsutsumi, Koji Inoue, Mutsushi Kawakita

| **Supplemental Figure 1.** The delta variation analysis of postoperative eGFR. (**a**) The multivariate analysis of variance for the time-dependent delta variation of eGFR between CRO-RAPN and CRO-LPN groups after matching. (**b**) Comparison of percentage of eGFR decrease at 3 and 6 months postoperatively between CRO-RAPN and CRO-LPN groups after matching.  **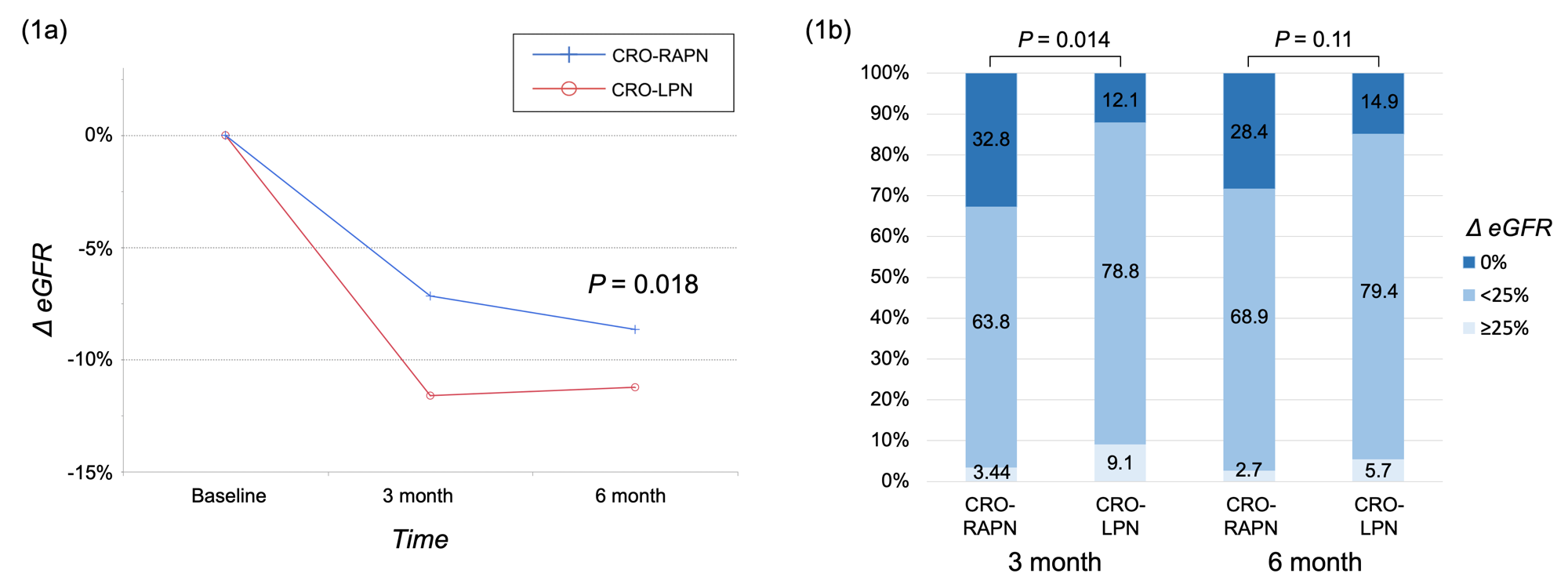**  **Supplemental Table 1.** Histopathological outcomes of the CRO-RAPN and CRO-LPN groups before and after propensity-score matching. | | | | | | | |
| --- | --- | --- | --- | --- | --- | --- | --- |
|  |  |  |  |  |  |  |  |
|  | **Before propensity-score matching** | | |  | **After propensity-score matching** | | |
| **Parameters** | **CRO-RAPN**^‡‡^ **group** | **CRO-LPN**^§§^ **group** | **p-value** |  | **CRO-RAPN group** | **CRO-LPN group** | **p-value** |
| **Number of patients** | **210** | **81** |  |  | **75** | **75** |  |
| Pathological stage, n (%) |  |  |  |  |  |  |  |
| T1a | 168 (81) | 60 (74) | 0.0012 |  | 61 (81) | 56 (75) | 0.092 |
| T1b | 19 (9) | 5 (6) |  |  | 7 (9) | 5 (7) |  |
| T2a | 5 (2) | 2 (2) |  |  | 1 (1) | 2 (3) |  |
| T2b | 5 (2) | 0 (0) |  |  | 0 (0) | 0 (0) |  |
| T3a | 1 (1) | 8 (10) |  |  | 0 (0) | 7 (9) |  |
| NA | 12 (6) | 6 (7) |  |  | 6 (8) | 5 (7) |  |
| Grade, n (%) |  |  |  |  |  |  |  |
| 3 | 22 (10) | 9 (11) | 0.060 |  | 6 (8) | 9 (12) | 0.048 |
| 2 | 134 (64) | 39 (48) |  |  | 50 (67) | 35 (47) |  |
| 1 | 39 (19) | 26 (32) |  |  | 12 (16) | 25 (33) |  |
| NA | 15 (7) | 7 (9) |  |  | 7 (9) | 6 (8) |  |
| Pathological type, n (%) |  |  |  |  |  |  |  |
| Clear cell | 173 (82) | 63 (78) | 0.71 |  | 58 (77) | 60 (80) | 0.83 |
| Papillary | 12 (6) | 7 (9) |  |  | 6 (8) | 5 (7) |  |
| Chromophobe | 9 (4) | 2 (2) |  |  | 4 (5) | 2 (3) |  |
| Sarcoma | 1 (1) | 1 (1) |  |  | 0 (0) | 1 (1) |  |
| Oncocytoma | 2 (1) | 1 (1) |  |  | 1 (1) | 1 (1) |  |
| Angiomyolipoma | 6 (3) | 4 (5) |  |  | 3 (4) | 3 (4) |  |
| Others | 7 (3) | 3 (4) |  |  | 3 (4) | 3 (4) |  |

^‡‡^cortical-renorrhaphy-omitting robot-assisted partial nephrectomy; ^§§^cortical-renorrhaphy-omitting laparoscopic partial nephrectomy.

| **Supplemental Table 2.** Patient characteristics in the CRO-RAPN and CRO-LPN groups before and after propensity-score matching with adjustment for warm ischemic time. | | | | | | | |  |
| --- | --- | --- | --- | --- | --- | --- | --- | --- |
|  |  |  |  |  |  |  |  | |
|  | **Before propensity-score matching** | | |  | **After propensity-score matching** | | | |
| **Parameters** | **CRO-RAPN**^‡‡^ **group** | **CRO-LPN**^§§^ **group** | **p-value** |  | **CRO-RAPN group** | **CRO-LPN group** | **p-value** | |
| **Number of patients** | **210** | **81** |  |  | **74** | **74** |  | |
| Median age, years (IQR^†^) | 67 (57–75) | 68 (54–76) | 0.79 |  | 67 (57–73) | 68 (54–76) | 0.74 | |
| Male sex, n (%) | 128 (61) | 50 (62) | 0.51 |  | 47 (63) | 48 (64) | 0.50 | |
| Median BMI^‡^, kg/m^2^ (IQR) | 23.9 (21.5–26.4) | 24.3 (21.8–25.9) | 0.96 |  | 24.3 (21.6–26.5) | 24.3 (21.9-26.0) | 0.55 | |
| ASA PS^§^ 3, or more, n (%) | 25 (12) | 8 (10) | 0.40 |  | 5 (7) | 7 (9) | 0.38 | |
| eGFR^¶^, mL/min/1.73m^2^ (IQR) | 67 (55–77) | 73 (56–85) | 0.040 |  | 68 (60–74) | 73 (56–85) | 0.11 | |
| KDIGO CKD^††^grade, n (%) |  |  |  |  |  |  |  | |
| Grade 1, or 2 (60 mL/min/1.73 m^2^, or more) | 143 (68) | 60 (74) | 0.55 |  | 57 (77) | 55 (74) | 0.92 | |
| Grade 3a, or 3b (30–59 mL/min/1.73 m^2^) | 58 (28) | 19 (23) |  |  | 15 (20) | 17 (23) |  | |
| Grade 4, or 5 (29 mL/min/1.73 m^2^, or less) | 9 (4) | 2 (2) |  |  | 2 (3) | 2 (3) |  | |
| Right side tumor, n (%) | 97 (46) | 41 (51) | 0.29 |  | 39 (53) | 39 (53) | 0.56 | |
| Tumor size, mm (IQR) | 30 (22–39) | 29 (23–37) | 0.84 |  | 30 (20–43) | 29 (23–37) | 0.79 | |
| Clinical T stage |  |  |  |  |  |  |  | |
| T1a | 159 (75) | 64 (79) | 0.59 |  | 51 (69) | 61 (82) | 0.15 | |
| T1b | 44 (22) | 16 (20) |  |  | 22 (30) | 12 (16) |  | |
| T2 | 7 (3) | 1 (1) |  |  | 1 (1) | 1 (1) |  | |
| R.E.N.A.L. nephrometry score, n (%) |  |  |  |  |  |  |  | |
| 4–6 (Low) | 95 (45) | 48 (59) | 0.047 |  | 40 (54) | 43 (58) | 0.77 | |
| 7–9 (Intermediate) | 89 (42) | 29 (36) |  |  | 28 (38) | 27 (37) |  | |
| 10–12 (High) | 26 (13) | 4 (5) |  |  | 6 (8) | 4 (5) |  | |
| Off-clamp procedure, n (%) | 13 (6) | 19 (23) | <0.001 |  | 12 (16) | 15 (20) | 0.34 | |
| Median warm ischemic time, minutes (IQR) | 16 (11–23) | 20 (16–28) | <0.001 |  | 18 (11-27) | 19 (11-24) | 0.54 | |
|  |  |  |  |  |  |  |  | |
| ^†^interquartile range; ^‡^body mass index; ^§^American Society of Anesthesiologists Physical Status classification; ^¶^estimated glomerular filtration rate; ^††^chronic kidney disease; ^‡‡^cortical-renorrhaphy-omitting robot-assisted partial nephrectomy; ^§§^cortical-renorrhaphy-omitting laparoscopic partial nephrectomy. | | | | | | | |  |
|  |  |  |  |  |  |  |  |  |

| **Supplemental Table 3.** Functional outcomes of the CRO-RAPN and CRO-LPN groups after adjustment for warm ischemic time during propensity-score matching. | | | |
| --- | --- | --- | --- |
|  |  |  |  |
|  | **After propensity-score matching** | | |
| **Parameters** | **CRO-RAPN**^§^ **group** | **CRO-LPN**^¶^ **group** | **p-value** |
| **Number of patients** | **74** | **74** |  |
| 6-month preservation rate of eGFR^‡^ |  |  |  |
| Median, % (IQR^†^) | 92 (86–100) | 90 (82–95) | 0.031 |
| ≥ 90%, n (%) | 47 (64) | 37 (64) | 0.068 |
| ≥ 80%, n (%) | 68 (92) | 63 (85) | 0.15 |
| ≥ 70%, n (%) | 74 (100) | 70 (95) | 0.060 |
| Median 6-month decrease of eGFR, mL/min/1.73 m^2^ (IQR) | 5 (0–9) | 6 (4–12) | 0.028 |
| Upstaging of chronic kidney disease, n (%) | 17 (23) | 19 (26) | 0.42 |
|  |  |  |  |
| ^†^interquartile range; ^‡^estimated glomerular filtration rate; ^§^cortical-renorrhaphy-omitting robot-assisted partial nephrectomy; ^¶^cortical-renorrhaphy-omitting laparoscopic partial nephrectomy. | | | |
